# Immunology of urinary tract infections

**DOI:** 10.3205/id000065

**Published:** 2020-05-12

**Authors:** José Antonio Ortega Martell

**Affiliations:** 1 Faculty of Medicine, Health Sciences Institute, Autonomous University of Hidalgo State, Pachuca, México

## Abstract

The urinary tract is constantly exposed to different microorganisms that colonize the gastrointestinal tract and the urinary tract is normally well prepared to resist infections by these microorganisms. This resistance to infection is mainly accomplished by the versatility of the immune system in the urinary tract, with both innate and adaptive immune responses. With the increasing knowledge of how the immune system works in the urinary tract and also the recognition of the virulence attributes of uropathogens, several potentially effective and tailored strategies to contain or prevent urinary tract infections have emerged.

## Summary of findings

- Immune response to uropathogens relay closely to the interaction of many molecules and cells that participate in both innate and adaptive immune responses.
- Urinary microbiome contributes to homeostasis in the immune response but colonization with uropathogens can be favored by intrinsic pathogen virulence factors and local damage at the lower or upper urinary tract.
- Defense to uropathogens can be successfully accomplished with molecules such as antimicrobial peptides, uromodulin, pentraxin and other local factors produced by the urothelium and also with secretory IgA produced by specific B lymphocytes.
- It is desirable that the immune response acts quickly to stop infection without causing greater damage and the balance of pro-inflammatory and anti-inflammatory mechanisms is key to avoid excessive damage to the urothelium and recurrent infections.

## Introduction

A clear understanding of the immune response in the urinary tract could be a very important tool for physicians to reduce recurrent infections in the context of multi-resistant bacteria. 

A great progress has been made both in understanding the basic immune mechanisms of urinary tract infections and in translating these findings to clinical therapies [1]. In this section, recent advances in our understanding of the urinary immune system and urinary tract infections are discussed, emphasizing the importance of the balance between the inflammatory response and the uropathogen persistence. 

## Immunology of the urinary tract

### Uropathogens

**Figure 1:**
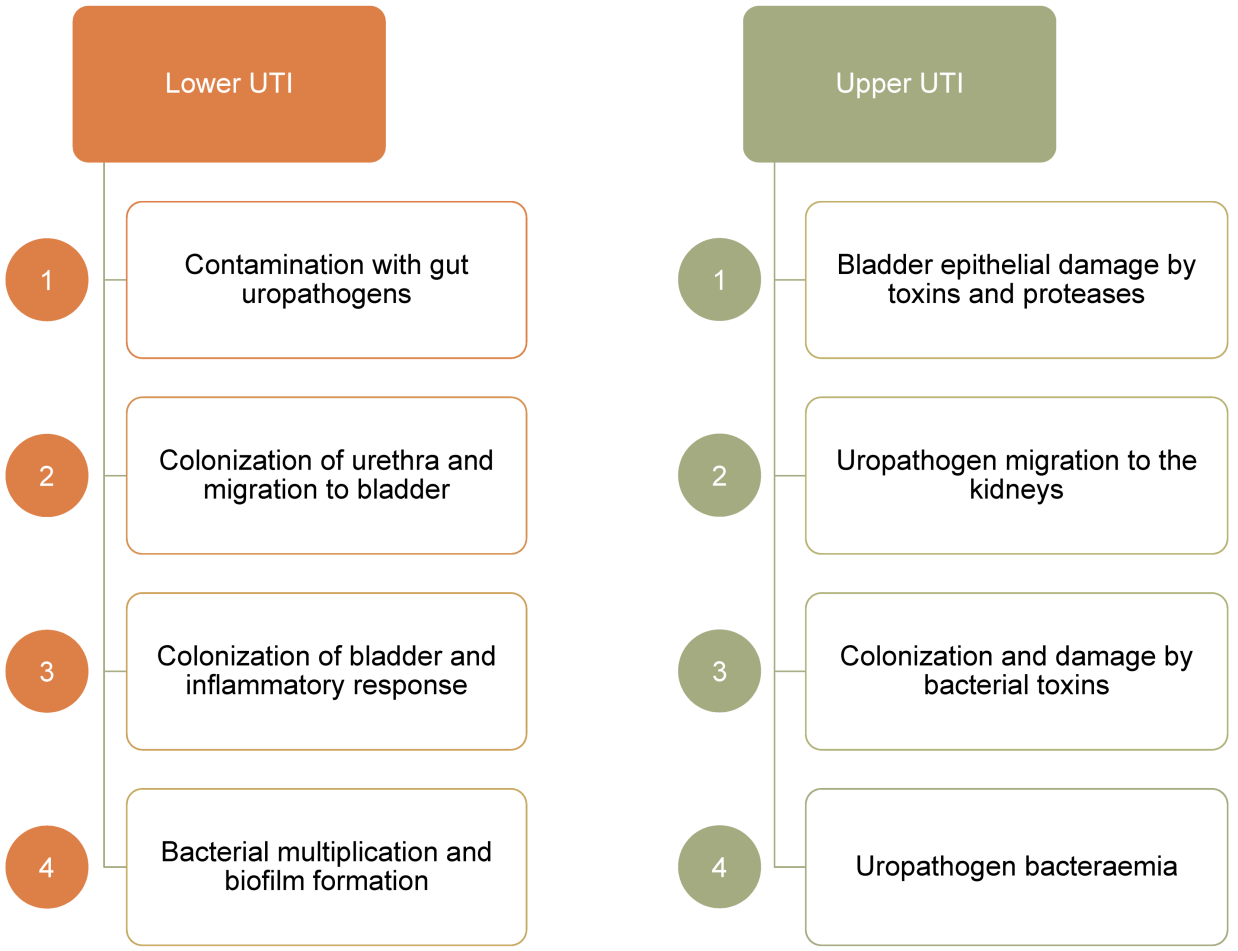
Urinary tract is a continuous road that can be colonized and damaged by uropathogens and uncontrolled inflammatory response.

The proximity of the urethra to the intestine makes colonization by uropathogenic *Escherichia coli* (UPEC) a frequent occurrence, particularly in catheterized patients. Ascending movement through the ureter can lead to inflammation and protease release causing kidney damage and hematogenic dissemination (Figure 1 (Fig. 1)) [2].

UPEC is the dominant organism in UTI, particularly in uncomplicated UTI. Other common uropathogens include *Klebsiella pneumoniae*, *Proteus mirabilis*, *Pseudomonas aeruginosa*, *Staphylococcus saprophyticus*, and *Enterococci faecalis* and *faecium*. All these uropathogens express virulence factors, which aid adhesion (e.g. pili, adhesins), nutrient release (toxins), immune evasion (capsules etc.), and iron acquisition (siderophores).

UPEC strains express a variable suite of virulence factors allowing adherence (fimbriae), invasion and defense against immune cells (endotoxins), movement and migration up the urinary tract (flagella), nutrition (iron receptors), and immune-system evasion (capsule) (Table 1 (Tab. 1)) [2]. UPEC counteracts urinary tract defenses and is able to avoid neutrophils by forming biofilms. Biofilms aid proliferation and colonization of the urethra and bladder by sacrificing the outer cells to protect the inner core. Intracellularly, UPEC can escape TLR4 mediated expulsion, manipulate lysosomes to impair their digestive capacity, and remain within the autophagosome membrane to avoid phagocytosis when expelled [3]. Notably, *Escherichia coli* found in the intestine tend to act as commensal organisms lacking the breadth of virulence factors found in UPEC strains [4]. One key determinant of UPEC virulence is the Ubi I gene, which is essential for the expression of type 1 pili, biofilm formation, and its pathogenesis [5].

#### Virulence factors

UPEC colonization of the urinary tract depends on its ability to bind host cells and tissues. Adherence also stimulates UPEC entry into host epithelial cells. The primary adherence factors encoded by UPEC are filamentous adhesive organelles known as pili or fimbriae. Common adhesive organelles elaborated by UPEC are type 1, P, S, and F1C pili encoded by the fim, pap, sfa, and foc operons, respectively [6]. Phase-variable expression of pilus genes within a single strain of UPEC can give rise to subpopulations expressing functionally distinct pili, increasing the probability of adherence to or invasion of host tissues and possibly broadening host specificity. Two of the most studied adhesive organelles are type 1 and P pili, which are encoded by many UPEC strains. 

#### P pili (fimbriae)

The expression of P pili is often associated with pyelonephritic UPEC isolates. A specific adhesin protein, called PapG, localized at the distal tip of the P pilus, mediates bacterial adhesion to host cells. Three types of the PapG adhesin have been identified (PapG I, II, and III) that recognize globotriasylceramide variants on the surface of target cells, particularly in the kidney [6]. 

#### Type 1 pili (fimbriae)

Type 1 pili are highly conserved and extremely common among both UPEC and commensal isolates and have come to be considered one of the most important virulence factors involved in the establishment of a UTI. Type 1 pilus adhesin FimH contains a binding pocket that recognizes mannose-containing host glycoprotein receptors [6] and mediates both bacterial adherence to and invasion of host cells, and contributes to the formation of intracellular bacterial biofilms by UPEC. The FimH adhesin can bind many host proteins, in particular, α3β1 integrin subunits expressed by host cells. β-1 integrins are surface adhesion molecules that link extracellular matrix proteins with the actin cytoskeleton, providing signaling conduits between the intra- and extracellular environments. Manipulation of integrins and downstream signaling cascades is a common mechanism by which pathogens gain entry into host cells [6].

#### ABU vs. UPEC

The majority of asymptomatic bacteriuria (ABU) cases are caused by *E. coli*. Asymptomatic bacterial carriage in the bladder resembles commensalism at other mucosal sites. Patients with ABU may carry the same strain for months or years without developing a disease response, leaving these commensal-like bacteria to successfully co-evolve with their hosts in a niche with little microbial competition. In epidemiological studies, ABU has been shown to protect against recurrent, symptomatic infection with more virulent strains [7]. Bacteria increase their fitness in new host niches by rapid adaptation to changing environmental conditions. They lose or gain genetic material and, through selection, new variants become fixed in the population. ABU strains evolve towards commensalism in human hosts, as defined by a reduction in overall genome size, inactivation of virulence genes and modifications of transcriptional regulators [8]. ABU isolates contain lower proportion of strains that showed functional type 1 and P fimbriae, motility on human urine plates, and hemolytic activity. The different response to UPEC and commensal-like ABU infections is epigenetically regulated by DNA/RNA methylation and histone acetylation [9]. The differential expression of prominent AMP genes shows that strong antimicrobial activities are induced by UPEC but not by ABU *E. coli* strains. These emphasize the importance of host epigenetic regulation of the innate immune response, allowing the discrimination of uropathogenic and commensal *E. coli* strains.

### Defense against uropathogens

As new and intriguing details of how uropathogens initiate infections and persist within the urinary tract have emerged, so has important information regarding how the immune system functions within the urinary tract [10]. 

#### Innate immunity

Recent studies have revealed the existence of a multifaceted innate immune response triggered by toll-like receptor 4 (TLR4) on superficial bladder epithelial cells directed at clearing infection by Gram-negative pathogens, and maybe a less important or a different role of the adaptive immunity. Though previously considered sterile, the bladder microbiome is increasingly thought to have a protective role alongside that of the urethra [11], [12]. Barriers to pathogenic bacterial colonization of the urinary tract include acidic pH and urine transport. The urothelium is the major constitutive barrier to infection in the urinary tract. It consists of mucus glycosaminoglycans – which retard pathogen adherence –, several layers of infection-resistant multinucleated umbrella cells, and glycoprotein plates called uroplakins. Apoptotic infected epithelial cells are released into the bladder lumen through exfoliation, reducing the bacterial load. Inner basement stromal cells producing new urothelium replace these apoptotic cells. 

**Figure 2:**
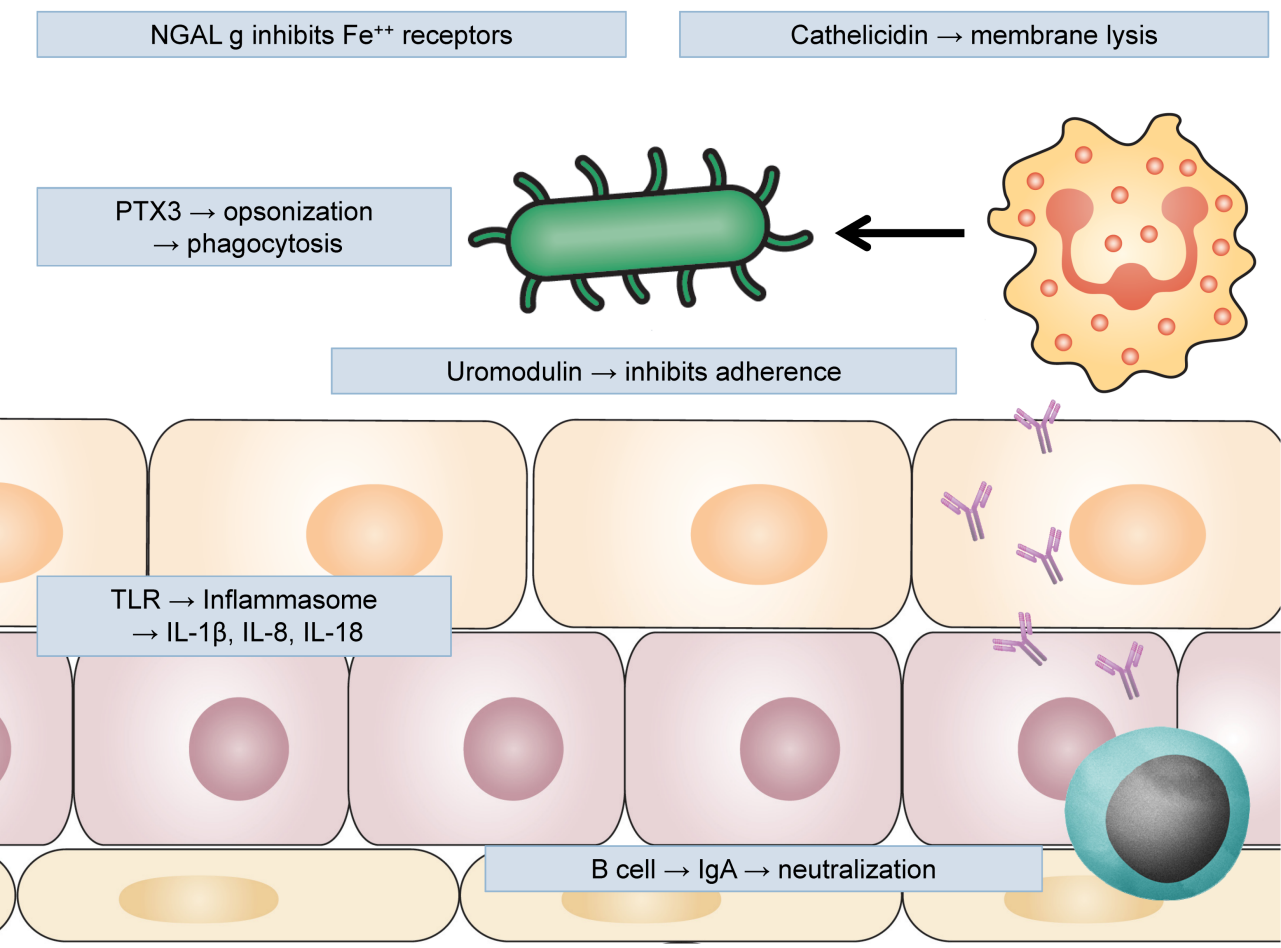
Uromodulin produced by urothelium inhibits adherence of UPEC; NGAL (neutrophil gelatinase-associated lipocalin) inhibits iron receptors blocking bacterial capacity to proliferate; cathelicidin and other antimicrobial peptides cause membrane lysis; pentraxin 3 (PTX3) opsonizes uropathogens and facilitates their phagocytosis by neutrophils and macrophages; epithelial cells activate inflammasome trough TLRs, producing pro-inflammatory cytokines; adaptive immune response also participates with B lymphocytes producing antigen-specific IgA neutralizing uropathogens.

Soluble factors, such as uromodulin, can inhibit UPEC adherence to epithelial cells. Another soluble factor, cathelicidin (LL-37), an antimicrobial peptide (AMP), forms an important component of the response to pathogens, and UPEC in particular, by targeting virulence factors. The neutrophil gelatinase-associated lipocalin (NGAL) protein binds bacterial siderophores limiting bacterial proliferation. Pentraxins (PTX) are an evolutionarily conserved protein family that can function as soluble toll-like receptors (TLRs). PTX3 is thought to bind bacterial surfaces, which leads to complement-mediated killing and increased uptake by phagocytes. Immune cells are also present in both the epithelium and interstitial compartments. In the upper urinary tract, dendritic cells, macrophages, neutrophils, and lymphocytes interact to defend against microorganisms. In the lower tract, mast cells, macrophages, neutrophils, and particularly natural killer (NK) cells act to combat colonization (Table 2 (Tab. 2), Figure 2 (Fig. 2)) [3].

In addition to their barrier function, epithelial cells express TLRs to respond to pathogens. Activation of urothelium-expressed TLR4/5 leads to the release of pro-inflammatory cytokines, AMPs, and several chemokines, which attract neutrophils from the bloodstream into the bladder lumen for phagocytosis (Table 3 (Tab. 3)). Both macrophage and NK-cells release cytokines to promote this process while mast cell derived factors (e.g. histamine) cause vasodilation to aid cell migration [3].

#### TLR4

TLRs are innate immune receptors that contribute to host defense by initiating an inflammatory response to conserved microbial patterns that are expressed in both invading pathogens and commensal microorganisms. TLR4 on leukocytes is activated by LPS on the surface of Gram-negative bacteria, which leads to the recruitment of the accessory molecules: myeloid differentiation primary response 88 (MyD88) or toll/interleukin 1-receptor domain – containing adapter inducing interferon β (TRIF), contributing to the induction of the transcription factor NF-KB (nuclear factor-κ-light-chain-enhancer of activated B cells) or interferon regulatory factor 3 (IRF-3), and the eventual elimination of the invading microbe. Although TLR4 has a signature role in host defense through its expression on immune cells, TLR4 is also expressed on the uroepithelium, where it regulates epithelial apoptosis and migration, and proliferation, and thus contributes to the pathogenesis of urinary tract infections, especially if TLR4 activation is not modulated [7]. 

#### Adaptive immunity and immunomodulation

Whereas the wide-ranging innate immune responses of the urinary tract are highly responsive to infections, the adaptive immune responses, particularly in the bladder, tend to be limited. UTIs that progress to the kidneys can lead to the production of antibodies specific for the infecting agent, but patients with infections limited to the bladder inexplicably fail to induce an antibody response. This apparent defect in the antibody response of the bladder could be a major reason for the remarkable recurrence of UTIs, especially following bladder infection. The underlying basis of the inability of the bladder to mount an adaptive response has been linked to increased local IL-10 production, as IL-10-deficient mice showed substantial antibody responses to bladder infection [10]. Mast cells are a major source of IL-10 in the bladder following bacterial infection. Although these secretory cells have an important role in initiating a vigorous immune response in the early phases of bladder infection, mast cells seem to reverse their activity approximately 6 hours post-infection by switching to IL-10 production to shut down this response. Mast cell-generated IL-10 can prevent the expression of co-stimulatory molecules on dendritic cells (DCs) and thereby limit their capacity to function as effective antigen-presenting cells when they traffic to draining lymph nodes. Hence, consistent with the role of mast cell-derived IL-10 in attenuating innate immune response, the inability of the bladder to mount an antibody response to bacterial infection could be a by-product of its attempt to prevent harmful adaptive immune responses to the contents of urine, as well as to facilitate the rapid regeneration of its epithelium following infection-induced damage.

**Figure 3:**
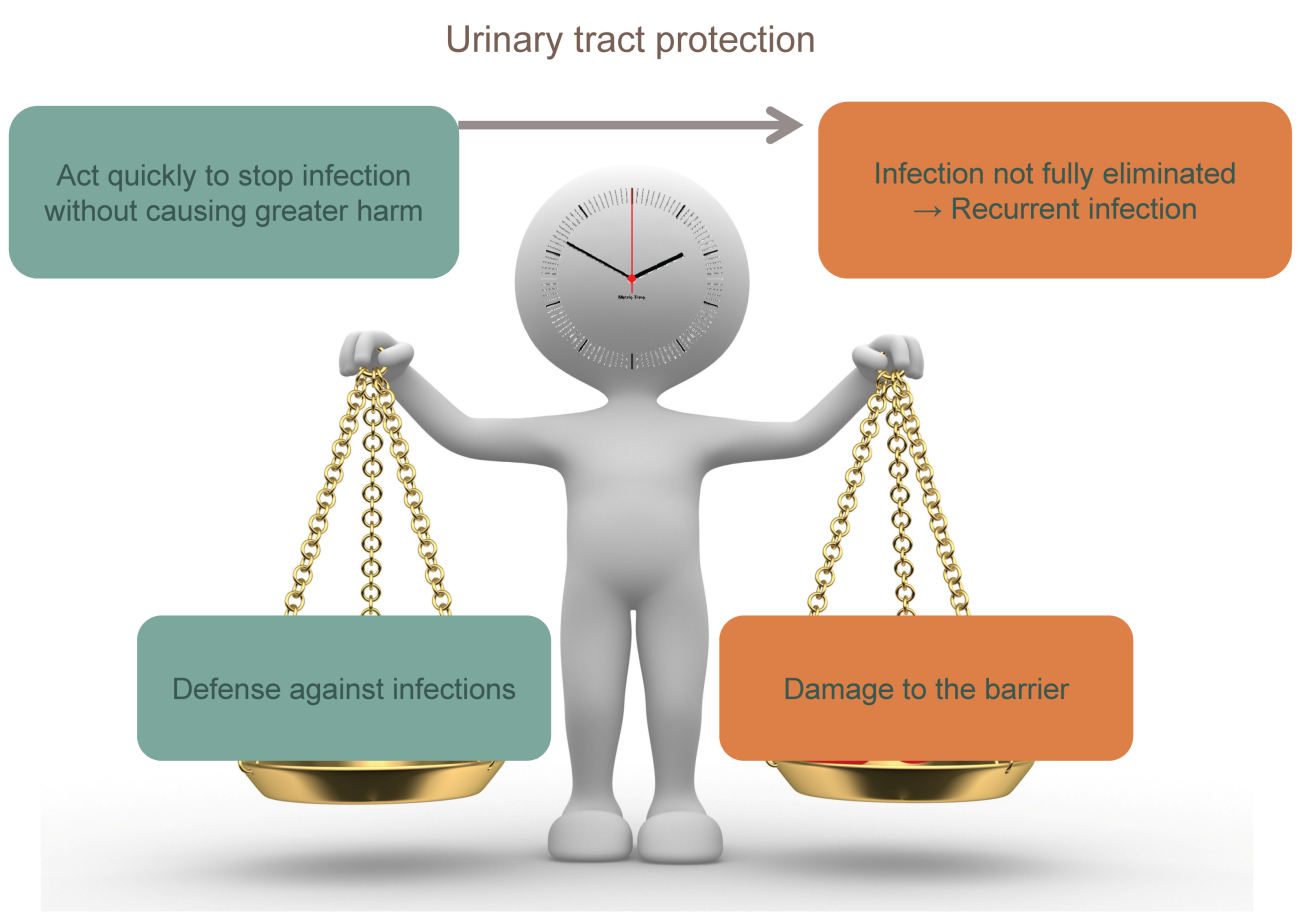
To obtain an adequate urinary tract protection, the immune system must act quickly to stop uropathogen invasion without causing greater harm. If this response is curtailed too soon in order to prevent more damage, infection could not be fully eliminated leading to recurrent infections.

As in all immune responses, the stimulus-dependent branches of the immune system in the urinary tract should balance between potency of response and excessive inflammation. An imbalance may result in persisting bacteria causing subsequent infection or inflammatory damage to the urothelium (Figure 3 (Fig. 3)). For example, TLR/PRR activation induces cell-specific inflammatory responses aimed at defense but is also associated with kidney disease [13]. In order to maintain balance, neutrophils are expelled in the urine to reduce inflammation. In the latter stage of infection, mast cells along with other regulatory cell populations take on an inhibitory role, keeping dendritic cells in an immature T-cell inhibitory state and reducing inflammation though IL-10 production (Table 4 (Tab. 4)). In addition, neutrophils are capable of producing anti-inflammatory meta-protease enzymes that induce urothelium restoration [13].

## Further research

As the nature of the immune responses in the urinary tract has become clearer, this knowledge could be useful to develop novel and more effective strategies for the prevention and treatment of urinary tract infections. Several of these strategies are targeted at boosting innate immune responses and some target adaptive immune responses.

## Conclusions

The physical barrier and the antibacterial actions of urinary secretions and resident cells lining the urinary tract constitute an integrated multi-layered defense system that protects against most prospective pathogens.

Due to the overriding need to retain or restore the epithelial barrier, innate as well as adaptive immune responses in the bladder are often prematurely curtailed, predisposing individuals to either chronic or recurrent infections, often by the same bacterial strain.

Identifying the nature of the defect in the urinary immune system – for example, through screening for presence or absence of certain biomarkers and examining for single-nucleotide polymorphisms – may become a key requirement in the diagnosis of UTIs. This information should allow for the future tailoring of treatments for individual patients so that it is not only appropriate but also optimal [14], [15].

## Note

This article is also to be published as a chapter of the Living Handbook „Urogenital Infections and Inflammations“ [16].

## Competing interests

The author declares that he has no competing interests.

## Figures and Tables

**Table 1:**
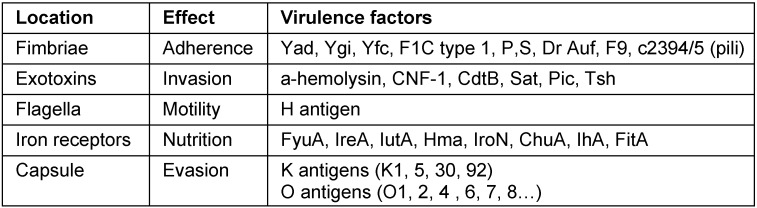
List of some virulence factors of uropathogenic *Escherichia coli* and their consequences during colonization and dissemination

**Table 2:**
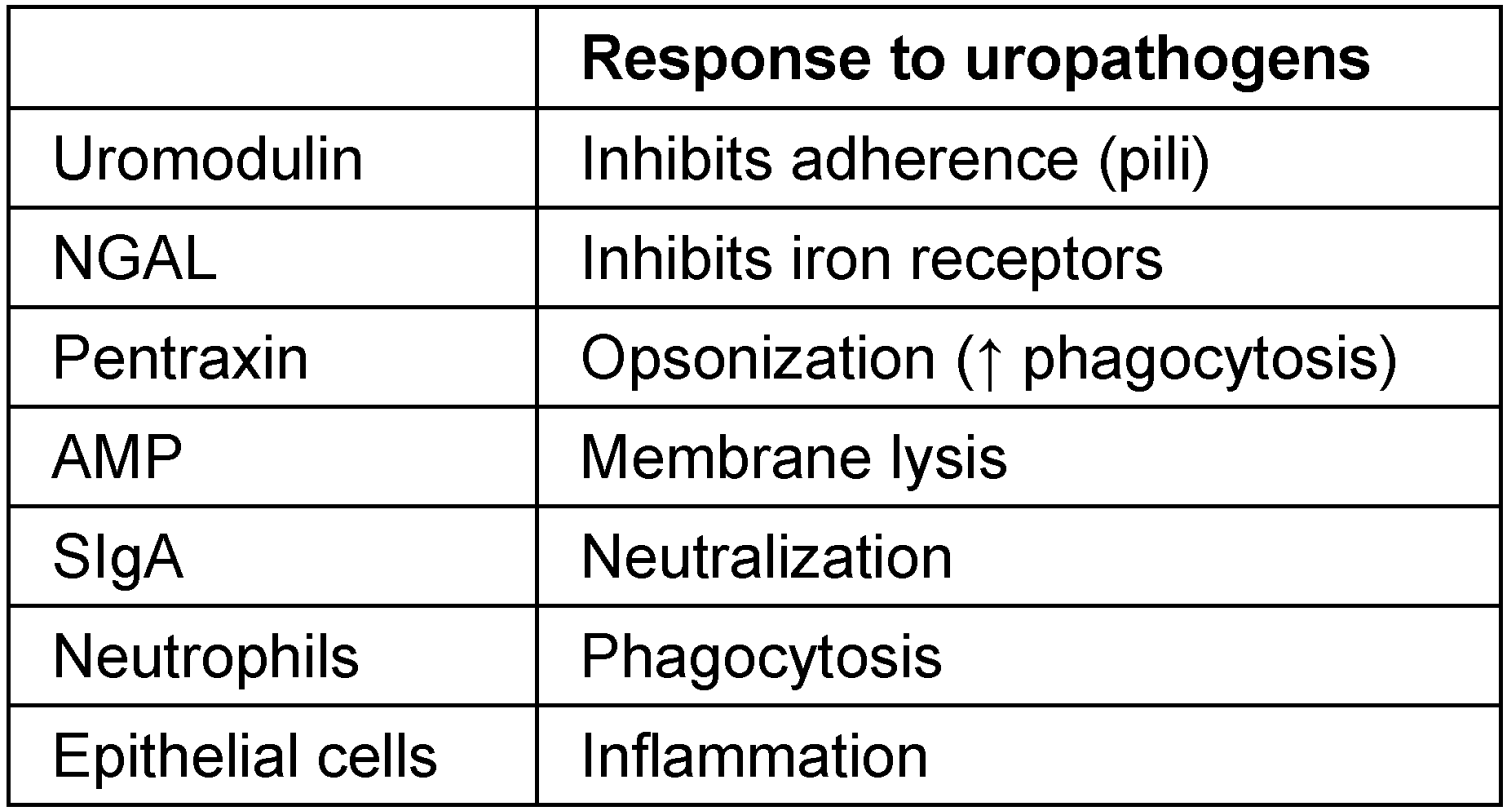
Partial list of representative soluble factors and cells that participate during the response to uropathogens

**Table 3:**
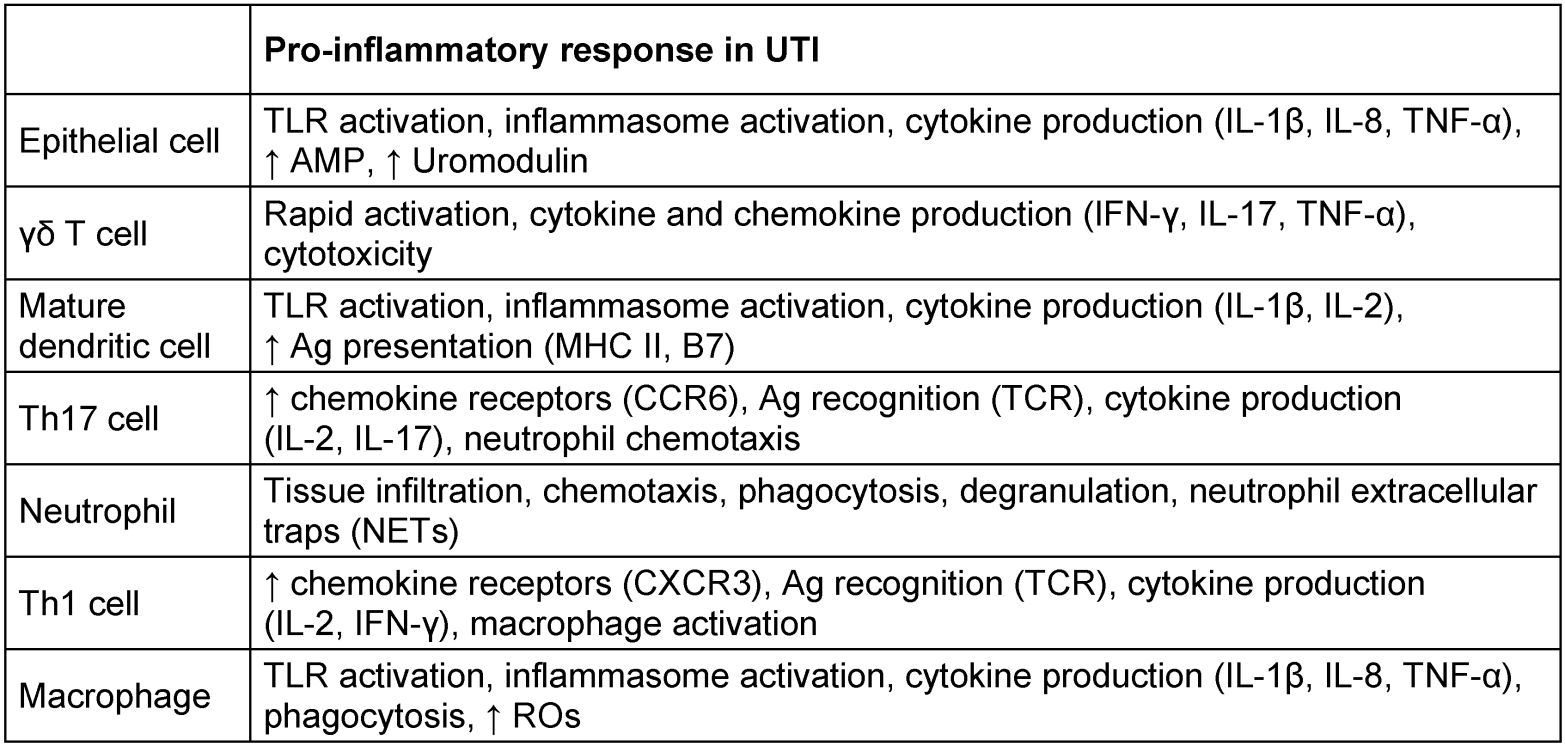
List of some pro-inflammatory cells with their molecules and mechanisms

**Table 4:**
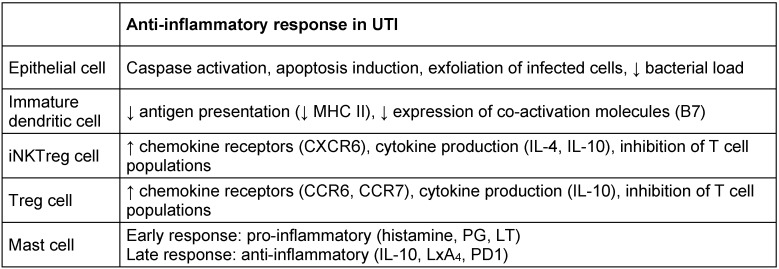
List of some anti-inflammatory cells with their molecules and mechanisms
